# Congenital Portosystemic Shunts in Dogs and Cats: Classification, Pathophysiology, Clinical Presentation and Diagnosis

**DOI:** 10.3390/vetsci10020160

**Published:** 2023-02-17

**Authors:** Alexandros O. Konstantinidis, Michail N. Patsikas, Lysimachos G. Papazoglou, Katerina K. Adamama-Moraitou

**Affiliations:** 1 Companion Animal Clinic (Medicine Unit), School of Veterinary Medicine, Faculty of Health Sciences, Aristotle University of Thessaloniki, 54627 Thessaloniki, Greece; 2 Laboratory of Diagnostic Imaging, School of Veterinary Medicine, Faculty of Health Sciences, Aristotle University of Thessaloniki, 54627 Thessaloniki, Greece; 3 Companion Animal Clinic (Surgery and Obstetrics Unit), School of Veterinary Medicine, Faculty of Health Sciences, Aristotle University of Thessaloniki, 54627 Thessaloniki, Greece

**Keywords:** Canine, diagnosis, Feline, portosystemic shunt, liver, symptoms

## Abstract

**Simple Summary:**

Congenital portosystemic shunts (CPSS) are abnormal vessels allowing communication between the splanchnic and systemic circulations. They are either extrahepatic (ECPSS) or intrahepatic (ICPSS), single or multiple. ECPSS is most commonly diagnosed in small and toy-breed dogs and cats, while ICPSS is most commonly seen in large-breed dogs. The clinical signs associated with CPSS, are due to hepatic encephalopathy (HE), and commonly involve the nervous system, gastrointestinal tract, and urinary tract. Neurological signs include depression, listlessness, ataxia, pacing, circling, head pressing, cortical blindness, seizures, and coma. Gastrointestinal signs, such as vomiting, diarrhea, anorexia, and ptyalism, occur in about 30% of dogs with CPSS. In cats with CPSS, ptyalism is a very common clinical sign, present in the vast majority of cases. Urinary signs include dysuria, stranguria, pollakiuria, and haematuria, and are associated with ammonium urate uroliths. The most common clinical pathology findings in dogs and cats with CPSS are mild to moderate microcytic normochromic nonregenerative anemia, elevated liver enzymes activities, hypoalbuminemia, decreased blood urea nitrogen (BUN), hypocholesterolemia, and hypoglycemia. Coagulation times are frequently found prolonged in affected dogs, but usually, they are not clinically significant. Urinalysis abnormalities include decreased urine specific gravity and ammonium biurate crystalluria. Liver function is evaluated by measurement of serum bile acids (BA) and fasting plasma ammonia (FA) concentrations. Both pre- and postprandial serum BA should be evaluated. Liver biopsy results include atrophy, decreased numbers of portal tributaries, and proliferation of arterioles and bile ductules. Survey radiographs are of limited value in the diagnosis of CPSS. Ultrasound is widely used for CPSS diagnosis due to its availability, but it is an operator-dependent technique. Computed tomographic angiography (CTA) has become the gold-standard technique for CPSS detection and characterization, as it is non-invasive, more accurate than ultrasound, and offers great surgical planning.

**Abstract:**

Congenital portosystemic shunts (CPSS) are abnormal vascular communications between the portal and the systemic circulation, bypassing the hepatic parenchyma and resulting in liver hypoplasia and hepatic insufficiency. Such connections develop in utero and persist postnatally. CPSS are among the two most common congenital vascular anomalies of the liver in small animals, along with primary hypoplasia of the portal vein without portal hypertension (PHPV without PH). CPSS can be extrahepatic (ECPSS), most commonly diagnosed in small and toy breed dogs and cats, or intrahepatic (ICPSS), most commonly seen in large breed dogs. Single ECPSS is the most common type encountered in both dogs and cats. Clinical signs of CPSS are non-specific and may wax and wane, while laboratory findings can raise clinical suspicion for CPSS, but they are also not specific. Definitive diagnosis will be established by evaluation of liver function tests, such as determination of fasting plasma ammonia (FA) levels, and pre- and postprandial serum bile acids concentrations, and diagnostic imaging. The purpose of this article is to review the definition, classification, pathogenesis, clinical presentation, and diagnosis of CPSS in dogs and cats, highlighted by the authors’ clinical experience.

## 1. Introduction

Congenital portosystemic shunts (CPSS) are anomalous vessels connecting the portal vein, or its tributaries, with the systemic circulation. These vessels permit venous blood, draining from the gastrointestinal tract, the pancreas, and the spleen to bypass the liver and enter directly into the systemic circulation. As a result, various toxins, including ammonia, that are absorbed from the gastrointestinal tract and normally metabolized or eliminated by the liver bypass it. Furthermore, various trophic factors from the GI tract and the pancreas, which would normally transport to the liver via the portal circulation, bypass it leading to parenchyma atrophy, decreased liver function and protein synthesis, and eventual hepatic failure. CPSS develops in utero and persists postnatally. CPSS and primary hypoplasia of the portal vein without portal hypertension (PHPV without PH) are the two most common congenital hepatic vascular anomalies of the liver in small animals. CPSS can be extrahepatic (ECPSS) or intrahepatic (ICPSS). Toy and small breed dogs usually have ECPSS, whereas large and giant breed dogs usually have ICPSS. CPSS occur also in cats, although with a much lower incidence. Single ECPSS is the most common type of CPSS encountered in both dogs and cats. The presenting history of dogs and cats with CPSS varies considerably. Clinical signs of CPSS are non-specific and may wax and wane and involve the central nervous system, the gastrointestinal and the urinary tract. Laboratory abnormalities include changes in the complete blood count, biochemistry, and urinalysis. Liver function tests, such as the determination of fasting plasma ammonia (FA) levels, and pre- and postprandial serum bile acids (BA) concentrations, and diagnostic imaging will confirm the diagnosis in dogs and cats suspected of having a CPSS.

The purpose of this article is to review the definition, classification, pathogenesis, clinical presentation, and diagnosis of CPSS in dogs and cats, highlighted by the authors’ clinical experience.

## 2. Anatomy of the Portal Venous System

**Figure 1:**
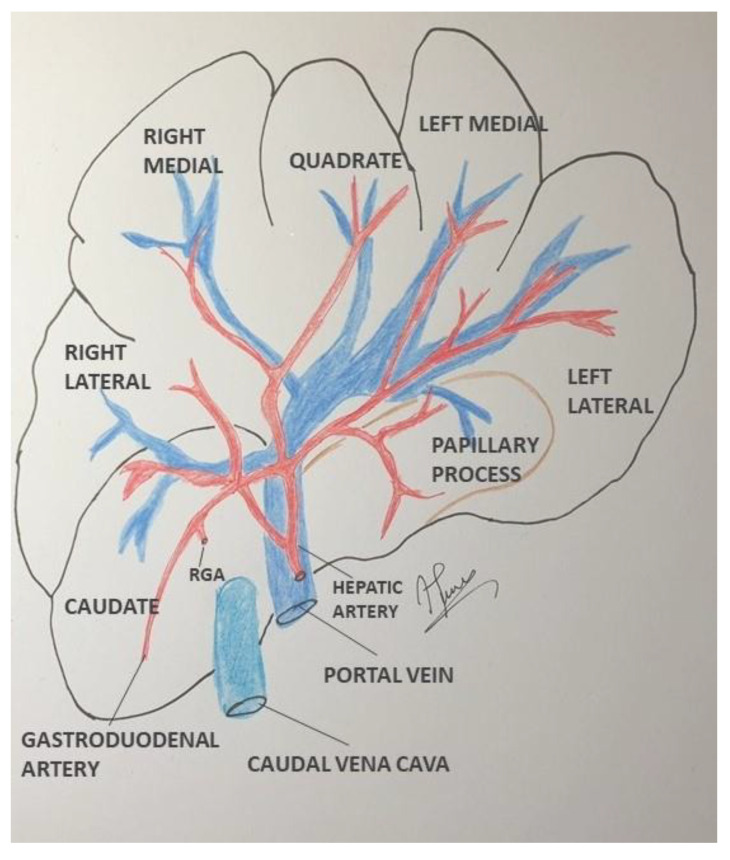
Schematic representation of lobar and divisional anatomy of the canine liver (visceral surface). RGA: Right Gastric Artery.

The hepatic vascular network consists of the portal vein, the hepatic artery, and the hepatic veins [1,2]. Portal circulation derives blood from the gastrointestinal (GI) tract, pancreas, and spleen via cranial and caudal mesenteric veins, splenic veins, and gastroduodenal and left gastric veins [1,2]. The hepatic blood flow represents 15–20% of cardiac output. About 80% of this flow is provided by the portal vein and the rest by the hepatic artery [3,4]. In dogs, the portal vein trunk is divided into right and left branches after entering the liver [1,5]. The right branch supplies the right division of the liver (right lateral and the caudate process of the caudate lobe). The left branch, bigger and longer than the right one, supplies the quadrate, the left medial, the left lateral as well as the papillary process of the caudate lobe and the right medial lobe via a central branch arising from the left branch of the liver [Figure 1]. In cats, the portal vein separates directly into left, central, and right branches [1]. The portal vein divides into smaller branches and portal blood enters the liver parenchyma via the portal triads [6]. Portal blood perfuses the liver through the sinusoid network mixing with blood from the hepatic artery and finally entering the hepatic veins and the caudal vena cava (CVC) [6].

## 3. Classification of Congenital Portosystemic Shunts

CPSS was reported as a vascular anomaly for the first time in 1949, and was described for the first time as a clinical entity in small animals in the 1970s [7,8,9]. Shunting of portal blood leads to the accumulation of toxins and waste products in the systemic circulation, as well as decreased delivery of essential trophic factors to the liver from the GI tract and pancreas, leading to hepatic hypoplasia and insufficiency. Since then, the etiology, epidemiology, diagnosis, and treatment of CPSS have been well described. CPSS can be ECPSS or ICPSS, with the former being the most common type. In recent studies, the morphology of ECPSS has been described in detail using computed tomographic angiography (CTA), intra-operative mesenteric portovenography, and gross anatomical appearance [10,11,12,13,14,15]. Many different types of ECPSS have been described in dogs and cats. The most common anatomical types are splenophrenic, splenoazygos, right gastrocaval, splenocaval, right gastrocaval with caudal loop, right gastrophrenic, left gastrophrenic, left gastroazygos, colonocaval and portocaval [Table 1] [14,15], whereas in cats the vast majority of ECPSS (92%) are spleno-caval, left gastro-phrenic and left gastro-caval [16]. ICPSS connect portal vein branches to the systemic circulation via hepatic veins or caudal vena cava and they result from the persistence of the ductus venosus or form during fetal development of hepatic sinusoids and portal vessels. ICPSS are classified as left-, right- and central-divisional [1,2,3]. They may open into the caudal vena cava or any hepatic vein branch. The morphology of ICPSS has been described in detail using CTA in recent studies [17,18,19,20,21,22]. In addition, new classification schemes of ICPSS based on their morphology have been proposed [Table 1] [18,19]. In dogs, left-divisional IHPSS is considered the most common variant [23,24]; however, in a recent retrospective study including 90 dogs with ICPSS, right-divisional ICPSS was the most common variant [25]. Of course, the variation in results may be due to differences in geographic variances and/or differences in recruitment or screening methods. Left-divisional IHPSS most likely represents the persistence of the fetal ductus venous, a vessel that diverts blood arriving at the portal sinus from the umbilical vein to the left hepatic vein in utero [26,27]. This vessel should normally close functionally within the first 6 days after birth and structurally by three weeks. Closure of the ductus venosus may be delayed in some breeds. In one study including 22 neonatal Irish Wolfhounds, the ductus venosus remained patent in 65% of puppies at 4 days of age and 23% at 6 days of age but was functionally closed in all puppies by day 9 [28]. ECPSS is also more common in cats. In a recent retrospective cohort study including 50 cats with ECPSS, the vast majority (40/50) had ECPSS and 10 had ICPSS [29]. The majority of ICPSS in cats are left-divisional and almost exclusively patent ductus venosus [24,30,31]. Typically, ICPSS shunt greater volumes of portal blood than ECPSS. In most cases, CPSS are usually single, although multiple concurrent CPSS have been reported [32].

## 4. Pathophysiology

Most of the clinical signs seen in dogs and cats with CPSS are related to hepatic encephalopathy (HE). HE is a neurologic dysfunction caused by hepatic disease, if more than 70% of hepatic function is lost, and/or portosystemic shunting [33,34]. A HE classification scheme for humans has been modified for veterinary patients [35]. According to this modified scheme, there are 3 broad types of hepatic disease resulting in HE, Type A: Acute liver failure without pre-existing disease, Type B: portosystemic shunting without intrinsic hepatocellular disease, and Type C: Cirrhosis, portal hypertension, and/or acquired shunt (including all acquired intrinsic hepatocellular diseases) [33,34]. In veterinary medicine, type B is the most common presentation of HE [36]. Clinical signs associated with HE vary and range from subtle nonspecific signs, such as a mild decrease in motility or apathy, to severe signs such as seizures and coma [33,34]. Furthermore, chronic HE (types B and C) may be persistent or episodic, and clinical signs may wax and wane. A scheme for grading the severity of HE in humans has been modified in veterinary medicine [Table 2] [33,34,37]. HE pathogenesis is complex and incompletely understood. The liver has a central detoxifying role with its capability of neutralizing many toxins absorbed from the GI tract and other byproducts of normal metabolism. Most of these toxins reach the liver through the portal circulation and go through the hepatic sinusoids these substances are effectively captured and detoxified by hepatocytes. If liver function is altered or portosystemic shunting occurs, the liver is unable to perform its vital functions including metabolism and detoxification. This results in the accumulation of toxic substances in the systemic circulation that severely affects the central nervous system. While the role of ammonia in HE is well characterized, a multitude of other toxins and factors are also implicated in its pathogenesis, such as oxidative stress, endogenous benzodiazepine-like ligands, astrocyte swelling, g-aminobutyric acid–like molecules, abnormal histamine and serotonin neurotransmission, endogenous opioids, neurosteroids, inflammatory cytokines, and potential manganese toxicity [38]. Several studies in humans highlight the role of ammonia and cerebral edema as major contributors to the development of HE [39,40]. Cerebral edema is partly due to the uptake of ammonia into astrocytes, where it is combined with intracellular glutamate to produce glutamine, which causes cellular swelling. However, the degree of encephalopathy is not well associated with blood ammonia levels [41]. The ammonia hypothesis alone does not fully explain the pathophysiology of HE. Infection and inflammation are also key drivers of HE. More precisely, HE is induced by rising ammonia levels in the presence of inflammatory mediators [42,43]. Even though the relationship between inflammation and HE in dogs needs to be better defined, several studies support the association between inflammation and HE in dogs with CPSS [44,45,46]. Furthermore, several factors that can potentially precipitate HE have been reported in dogs and cats [Table 3] [33,34,45]. It is very important to identify and treat any precipitating factors and to provide general supportive care (e.g., low protein diet, antibiotics, lactulose, etc.) to dogs and cats with HE.

Due to the shunt, portal blood bypasses the liver and, as a result, it does not develop normally. Shunting of portal blood results in liver hypoplasia with characteristic histopathological lesions (please see the Histopathology section) and impaired liver function [47]. Liver dysfunction results in laboratory findings such as hypoalbuminemia and hypoglycemia, as the liver is the only source of albumin, and plays a crucial role in maintaining glucose homeostasis.

The liver has a major role in coagulation, as it produces and clears most of the factors that regulate procoagulation, anticoagulation, and fibrinolysis. Several studies have examined coagulation status in dogs with CPSS documenting both bleeding and thrombotic tendencies, however, spontaneous bleeding or thrombosis is very rare [48,49].

## 5. Signalment

CPSS can be seen in any dog, but more frequently is presented in purebred dogs [50,51]. ECPSS are usually seen in small purebred dogs such as Yorkshire terriers, Maltese, Pugs, and Miniature Schnauzers [1,52,53], while ICPSS are seen ordinarily in large breed dogs, such as Labrador and Golden Retrievers, German Shepherd Dogs, Irish Wolfhounds, and Doberman Pinchers [54,55]. Less commonly, CPSS has been also reported, in domestic shorthair cats and purebreds including Siamese, Himalayans, Burmese, and Persians [3,30,56,57,58,59,60]. Predisposed breeds may differ from country to country due to different genetic or environmental characteristics of the population [53,58]. Interestingly, the canine breed may even affect the morphology of ECPSS, although there are contradictory findings between studies [53,58]. Furthermore, canine breeds with no predisposition to CPSS are more likely to have CPSS of unusual morphology or inoperable shunts compared to predisposed breeds [58]. Left-divisional shunts, due to the persistence of the fetal ductus venous, are considered hereditary in the Irish Wolfhounds [61,62,63]. Most dogs and cats with CPSS are usually diagnosed at less than 1 to 2 years old, although some cases may be older animals (5 or >10 years old) [30,56,60,64,65].

The vast majority of dogs with CPSS present at the authors’ veterinary teaching hospital are ECPSS (unpublished data). Less than 10% of the total CPSS cases are ICPSS. Worldwide geographical variation in the prevalence and/or morphology of CPSS probably exists. Maltese, Yorkshire terriers, Pug, and Shih Tzu with ECPSS are overrepresented also at the authors’ clinic. The number of cases diagnosed has increased over time. Interestingly, the number of dogs with ECPSS diagnosed at >5 or even >10 years old has also significantly increased over time; they are usually presented without the typical clinical signs of HE. Cases of cats with CPSS are rare.

## 6. Clinical Signs/Physical Examination

Clinical signs associated with CPSS may wax and wane or be intermittent. The central nervous system, as well as the GI and urinary tract, are mostly affected. Central nervous system signs result mainly from HE and possibly from hypoglycemia. Classic HE signs comprise seizures, behavioral changes, lethargy, ataxia, circling, head pressing, episodic central blindness, disorientatiοn, pacing, seizures, coma, and aggression [1,51,65]. Signs of HE can vary from very mild to extremely severe, or wax and wane. A large portion of dogs with CPSS does not manifest the classic symptoms of HE and owners describe them as quiet, depressed, or lethargic. Signs of encephalopathy, which worsen after a meal, are particularly suggestive of HE. However, there is no clear relationship between meals and HE signs, as only 30–50% of patients exhibit a correlation between clinical signs with food consumption [65,66]. In a retrospective study, 68% of dogs undergoing surgical attenuation of a single CPSS had preoperative neurological abnormalities [67]. Most cats (93–100%) are presented with neurological clinical signs due to HE [31].

GI signs, related to CPSS are usually mild, and non-specific and include intermittent vomiting, diarrhea, anorexia, weight loss, and melena [51,68]. GI hemorrhage has been reported both pre- and post-operatively in dogs with ICPSS and has been related to long-term morbidity and mortality [54]. Hypergastrinemia and increased gastric acid production, due to decreased hepatic clearance of gastrin and/or increased gastrin release stimulated by elevated serum bile acid (BA) concentrations, abnormal blood flow, hypoprostaglandinemia, poor mucosal integrity, and abnormal mucus production have been proposed as possible mechanisms for GI hemorrhage [3]. Upper GI endoscopy pre-operatively, documenting the GI hemorrhagic lesions, and administration of proton-pump inhibitors pre- and post-operatively reduce the incidence of this complication [54,69]. Ptyalism/hypersalivation is a very common clinical sign in cats with CPSS possibly associated with HE [3,70,71].

Hematuria, dysuria, stranguria, polyuria, pollakiuria, and urinary obstruction are common clinical signs in patients with CPSS [65,71,72,73,74,75,76,77]. Decreased urea production, increased ammonia excretion, and decreased uric acid metabolism may result in ammonium biurate crystals and stone formation and predisposition to bacterial urinary tract infections in dogs and cats with CPSS [65]. Polyuria/polydipsia is a common clinical finding in many patients with CPSS mainly due to deranged neuroendocrine functions [51,78].

Poor growth or lower body condition score, fever, and episodic weakness are other possible findings in dogs and cats with CPSS [56,71,75,77,79]. Portal hypertension (PH) is not present in CPSS, and patients do not develop ascites unless they have profound hypoalbuminemia due to GI bleeding, hepatic insufficiency, or severe dietary protein restriction. Finally, copper-colored irises are reported in a variable number of cats with CPSS but its etiology remains undetermined [56,70,71,77].

The severity of clinical signs may vary and can depend on the morphology of the CPSS. Portoazygous or portophrenic ECPSS may be associated with less severe clinical signs and diagnosed later in a dog’s life [80], probably because of partial compression of the ECPSS by the diaphragm during respiration and gastric distension following a meal [80,81].

According to the author’s experience, the majority of dogs with CPSS presented to their hospital have stunted growth and CNS signs due to HE, which may be intermittent or wax and wane with/or without GI signs. As others have reported previously, only a small number of dogs have the classic post-prandial HE signs. Very few dogs present only with lower urinary signs due to ammonium urate uroliths, which comes in agreement with previous studies.

## 7. Diagnosis

A combination of history and physical examination, clinicopathologic, especially liver function tests, and diagnostic imaging findings, will confirm the diagnosis of CPSS.

### 7.1. Hematology

In CPSS, possible hematologic findings include non-regenerative, microcytic, and normochromic anemia [70,77,82,83]. Microcytic anemia is more common in dogs than cats with CPSS and usually resolves after surgical attenuation but still, the cause remains unknown. Studies connect microcytosis to defective iron metabolism rather than iron deficiency [83,84]. However, a recent study found no association between dysregulated production of hepcidin and anemia in dogs with CPSS [85]. Leukocytosis is variably present possibly due to inadequate clearance of bacteria and/or endotoxins from the portal system resulting in bacteremia and/or endotoxemia and has been associated with a poor outcome [51,65,86,87].

### 7.2. Coagulation Profile

The liver plays a key role in hemostasis as most pro- and anticoagulant factors, as well as the pro- and antifibrinolytic factors, are synthesized by hepatocytes [88]. Most of these factors are also cleared by the liver. Hemostasis can be profoundly altered by liver disorders. Several studies have examined coagulation in dogs with CPSS and discovered changes in serum concentrations of both pro- and anticoagulant proteins [48,49,89,90]. Prolonged prothrombin time (PT) and activated partial thromboplastin time (aPTT), lower platelet counts, and deficiencies of procoagulant factors (II, V, VII, and IX) have been found, suggesting that dogs with CPSS may be hypocoagulable [49,89,90,91]. However, decreased antithrombin and protein C activities and increased factor VIII activity, and von Willebrand factor concentration suggests that dogs with CPSS may be hypercoagulable [49,89]. Furthermore, a recent study of thromboelastography analysis also found a tendency of dogs with CPSS to be hypercoagulable (as evaluated by G value), but none of the dogs included had evidence of bleeding or thrombosis [48]. Interestingly, the presence of a hypercoagulable state (high G values) was 40 times more likely to be associated with clinical hepatic encephalopathy [48]. However, despite the coagulation disorders reported, portal vein thrombosis and spontaneous bleeding are very rare [49,87,92].

Data regarding coagulation in cats with CPSS are limited. Tzounos et al. investigated changes in coagulation parameters in cats with CPSS and found increased PT and aPTT in the majority of the cases included (87.5% and 68.8%, respectively) [93]. However, none of the cats included demonstrated spontaneous bleeding or post-surgically complications associated with bleeding.

### 7.3. Serum Biochemistry

Serum biochemical abnormalities in dogs and cats with CPSS can vary. A mild to moderate increase in serum liver enzyme activity (alkaline phosphatase, ALP; alanine aminotransferase, ALT; aspartate aminotransferase, AST) is commonly reported. Hypoalbuminemia, hypoproteinemia, hypocholesterolemia, and low urea concentration due to reduced hepatic synthesis may be encountered. Hypoglycemia can be seen as a result of decreased hepatic glyconeogenesis and glycogen storage. Low serum creatinine and urea concentration can occur as a result of an increased glomerular filtration rate [70,77,78].

### 7.4. Urinalysis

**Figure 2:**
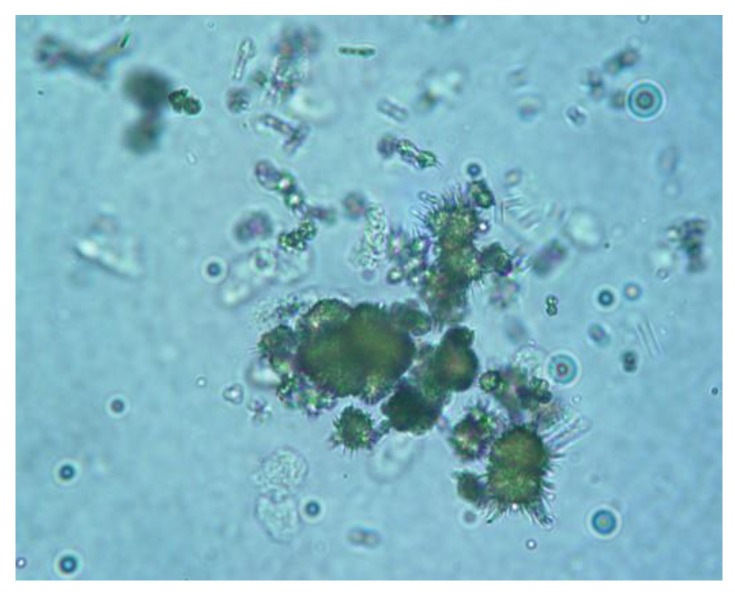
Ammonium biurate crystals in the shape of a thorn apple in the urine sediment of a dog with an extrahepatic congenital portosystemic shunt (400×). (Courtesy of Prof. Mathios E. Mylonakis, Companion Animal Clinic, School of Veterinary Medicine, Aristotle University of Thessaloniki).

Common urinalysis findings in dogs and cats with CPSS have decreased urine specific gravity and ammonium biurate crystalluria [Figure 2] [72,73,94,95,96]. Low urine specific gravity is the result of the poor medullary concentration gradient due to the decreased urea production in the hepatic parenchyma or HE and associated psychogenic polydipsia. Ammonium biurate crystalluria is reported in up to 57% of dogs, and 42% of cats with CPSS [70,72,73,74,77,96].

### 7.5. Liver Function Tests

Since history and clinical signs associated with CPSS may be non-specific or vary, liver function tests with high sensitivity and specificity are required for further investigation. Different tests to identify CPSS have been proposed.

Measurement of serum BA concentration is the most common liver function test used for diagnosis of CPSS in dogs and cats as well as for monitoring response to therapy. Increased serum BA concentration results from the shunting of reabsorbed BA into systemic circulation. To obtain maximum sensitivity (100%) of serum BA measurements both a 12-h fasting and a postprandial blood sample should be taken in dogs and cats with suspected CPSS (serum BA stimulation test), even though some studies report sensitivity up to 100% of a single serum BA postprandial sample [97,98,99,100,101]. Patients should be fed a high protein, moderate fat density meal to stimulate gallbladder contraction after obtaining the preprandial sample. However, increased serum BA concentration results not only from CPSS or acquired portosystemic shunts but also from other hepatic vascular anomalies or diseases associated with intra- or extra-hepatic cholestasis [102,103,104]. Clinically healthy Maltese dogs can have increased serum BA without evidence of hepatocellular dysfunction [105]. Dogs that are already on ursodeoxycholic acid should discontinue treatment for at least 72 h before measurement of serum BA, as long-term administration of ursodeoxycholic acid to healthy dogs may increase fasting serum BA values [106].

Plasma FA measurement is another liver function test used for the evaluation of hepatic insufficiency including CPSS. Plasma ammonia is mainly derived from dietary protein, and secondary from GI mucosa, GI hemorrhage, and kidneys. Ammonia is then delivered to the liver via the portal vein, where is converted to urea through the urea cycle by the hepatocytes. Increased plasma FA levels are usually found in dogs and cats with CPSS; however, false negative results may also be obtained [70,75,77,101]. Other possible causes of increased plasma FA concentrations are hepatic or urea cycle enzyme insufficiency [107,108]. The sensitivity of plasma FA concentration in the diagnosis of CPSS in dogs varies from 81% to 100% [65,96,109,110]. Moreover, dogs and cats with suspected CPSS can be challenged to determine their ability to clear ammonia. For this purpose, measurement of 6-h postprandial plasma ammonia concentrations and oral or colonic ammonia tolerance tests are employed. In the postprandial ammonia test, plasma ammonia concentrations are measured before (24 h fasting) and 6 h after feeding the 25% of the daily metabolizable energy requirements which may increase the test sensitivity to 91% [109]. In an oral or colonic ammonia tolerance test, ammonia concentration is measured in a fasted dog before and 30-min after the challenge. More specifically, ammonium chloride (NH4Cl) is administered orally using an orogastric tube or capsule (100 mg/kg; maximum of 3 g) or a high colonic infusion (2 mL/kg of a 5% solution infused through a tube inserted 20 to 35 cm into the colon) [1]. In a recent study, the rectal ammonia tolerance test had a sensitivity and negative predictive value of 100% for CPSS diagnosis [111]. However, an ammonia tolerance test can lead to exacerbation of HE and so is rarely performed [107,112]. Transient hyperammonemia due to urea cycle enzyme deficiency has been documented in young Irish Wolfhounds without CPSS [108,113].

The authors typically measure both serum BA and plasma FA on all dogs with suspected CPSS. More specifically, the authors perform both a serum BA stimulation test and a postprandial ammonia test, as well as CBC, full serum chemistry panel, and urinalysis to support CPSS diagnosis and exclude all other possible diseases leading to similar signs and included in the list of differential diagnosis.

Protein C is a vitamin K-dependent short-lived protein that is activated by thrombin and found in plasma [114]. Protein C is synthesized in the liver and is important for the maintenance of hemostatic balance, protects against thromboembolism, and regulates inflammation and apoptosis [115]. Protein C is used as a clinical biomarker for hepatic function and hepatoportal perfusion. In healthy dogs, protein C activity is 70% or greater, while its activity is decreased in a variety of hepatobiliary diseases, including CPSS [89,91,116]. In a very recent study, Sunlight et al. (2022) reported decreased protein C activity in dogs with ICPSS, that increased in the majority of dogs after successful percutaneous transvenous coil embolization [116]. In addition, Toulza et al. (2006) reported reduced protein C activity in 98% of dogs with CPSS compared to 30% in those with primary hypoplasia of the portal vein without PH [89]. This finding suggests that protein C could help differentiate between CPSS and primary hypoplasia of the portal vein without PH in addition to other blood tests. However, decreased protein C activity is reported in a variety of conditions including hepatic failure, sepsis, and congestive heart failure [117,118,119].

## 8. Histopathology

The histopathologic changes of the liver seen in CPSS reflect the portal vein hypoperfusion. Absence or portal vein hypoplasia, arteriolar proliferation or duplication, hepatocellular atrophy (lobular), bile duct proliferation, lipidosis, and lipogranulomas formation are common findings [47,120,121,122,123,124]. Smooth muscle hypertrophy, increased lymphatics around central veins, Ito and Kuppfer cell hypertrophy, hemosidirosis, and hepatic fibrosis or necrosis and inflammation have also been reported [23,57,120,121,122,123]. The severity of histopathologic changes is probably correlated to the amount of shunting blood and may vary between liver lobes depending on the shunt site [47]. Fibrosis, biliary hyperplasia, and necrosis have been associated with poor prognosis [3,123]. Other studies report that histologic lesions in CPSS dogs, either with ECPSS or ICPSS, are not correlated with survival times and prognosis [121,125]. Surgical attenuation of shunts does not result in the improvement of histopathologic lesions [120,124].

## 9. Diagnostic Imaging

**Figure 3:**
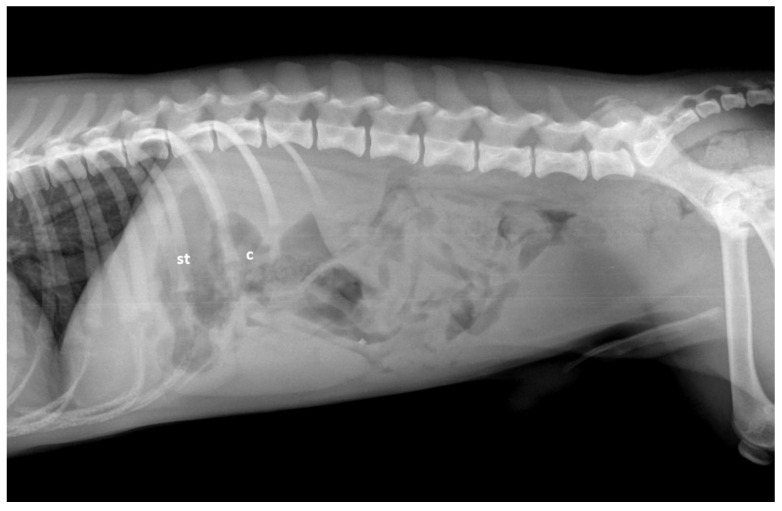
Lateral abdominal radiograph in a dog with a congenital extrahepatic portosystemic shunt. There is a cranial displacement of the stomach (st) and transverse colon (c) suggesting the presence of microhepatica.

Radiographs provide very limited information on canine hepatic vascular disorders. Possible findings indicative of CPSS include microhepatica and bilateral renomegaly [Figure 3] [126]. Ammonium biurate calculi are usually radiolucent, except when they contain additional calcium salt or struvite deposits.

**Figure 4:**
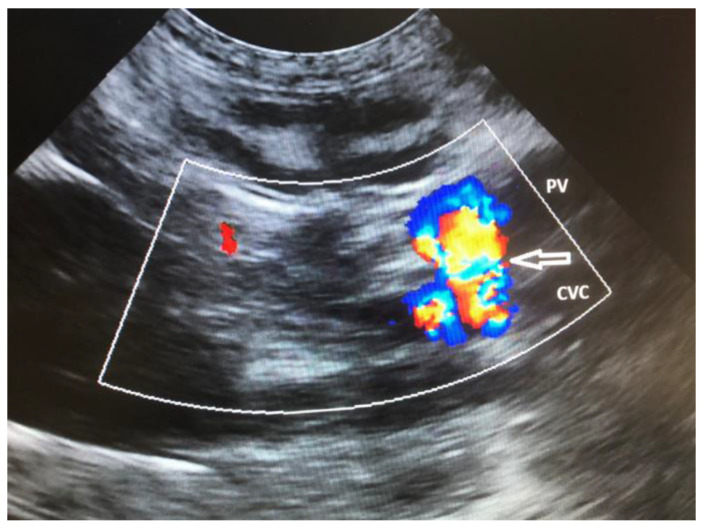
Sagittal plane ultrasound color flow Doppler image of the cranial abdomen in a dog showing the presence of an extrahepatic communication (arrow) of the portal vein (PV) with the caudal vena cava (CVC). There is turbulent blood flow within the shunt vessel indicated by the mosaic pattern of colors.

**Figure 5:**
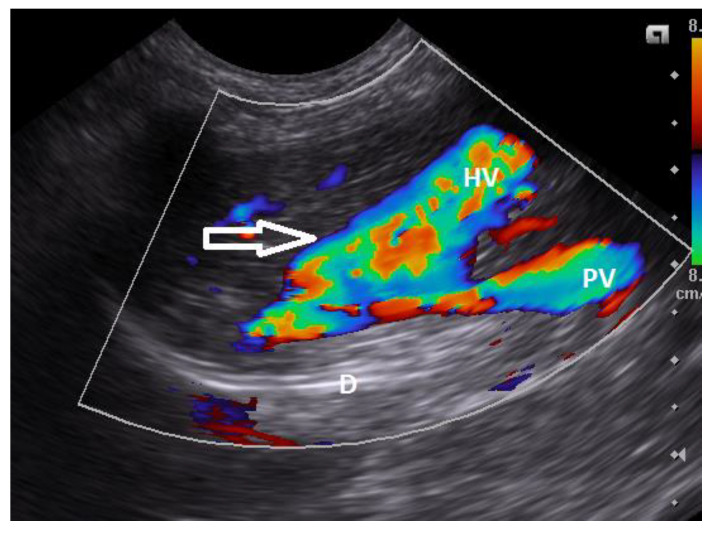
Oblique plane ultrasound color flows Doppler image of the right liver lobes in a young dog with an intrahepatic congenital portocaval shunt. There is an intrahepatic connection (arrow) of the portal vein branch (PV) with an anomalous dilated hepatic vessel (HV). (D: Diaphragm).

Ultrasonography (US) is the most commonly used technique for the detection of CPSS in dogs and cats due to its availability [56,76,126,127,128]. The US is a rapid, noninvasive technique that does not require general anesthesia. However, is an operator-dependent technique. Common findings are microhepatica, reduced hepatic and portal vein branch visibility, presence of an anomalous vessel or enlarged kidneys, and reduced portal vein/aorta ratio in dogs with ECPSS [30,56,60,76,128]. Turbulence in blood flow in the region of the CPSS insertion to the systemic venous system may also be identified. Diagnosis of CPSS requires imaging of the anomalous vessel from its origin to its termination [36,129]. Szatmári et al. (2004) described a protocol for systematic US evaluation of the portal system in dogs [129]. US drawback is the considerable variation in the reported accuracy for detection of CPSS (sensitivity 74–95%, specificity 67–100%), and therefore failure to locate a CPSS in US does not rule out the possibility of its presence [56,127,128,130]. ICPSS along with their morphology and hepatic division can be detected with US [Figure 4 and Figure 5 ], which sensitivity and specificity may reach 100% and they are more likely to be found compared to ECPSS due to their predictable location within the hepatic parenchyma [76,128,130].

**Figure 6:**
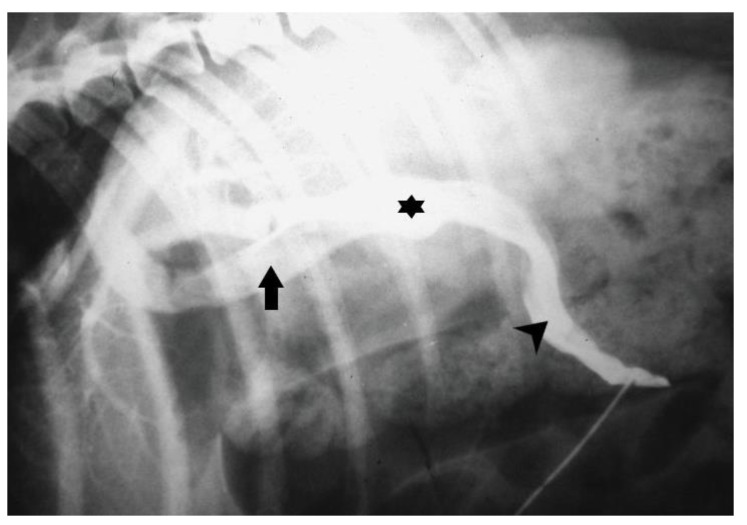
Intraoperative mesenteric portogram depicting an intrahepatic portocaval shunt (asterisk) (arrow: caudal vena cava; arrowhead: portal vein).

Mesenteric portovenography is another imaging technique for the detection of CPSS that currently is less commonly used due to the availability of other non-invasive techniques, like US and CTA. Mesenteric portovenography requires general anesthesia; a midline laparotomy is performed and a catheter is inserted into the jejunal vein. Water soluble, sterile, radiopaque contrast medium is injected into the vein as a bolus (2 to 4 mL/kg) [131]. Fluoroscopy or radiography is used for the detection of CPSS after contrasting medium injection. The sensitivity of mesenteric portovenography has been reported to range from 85% to 100% [Figure 6]. The same jejunal catheter can be used for the measurement of portal pressures intraoperatively. However, the invasive nature of this technique, the need for mobile radiographic facilities, the variation in the expected result, and the lack of presurgical planning have led to its withdrawal from the investigation of CPSS.

**Figure 7:**
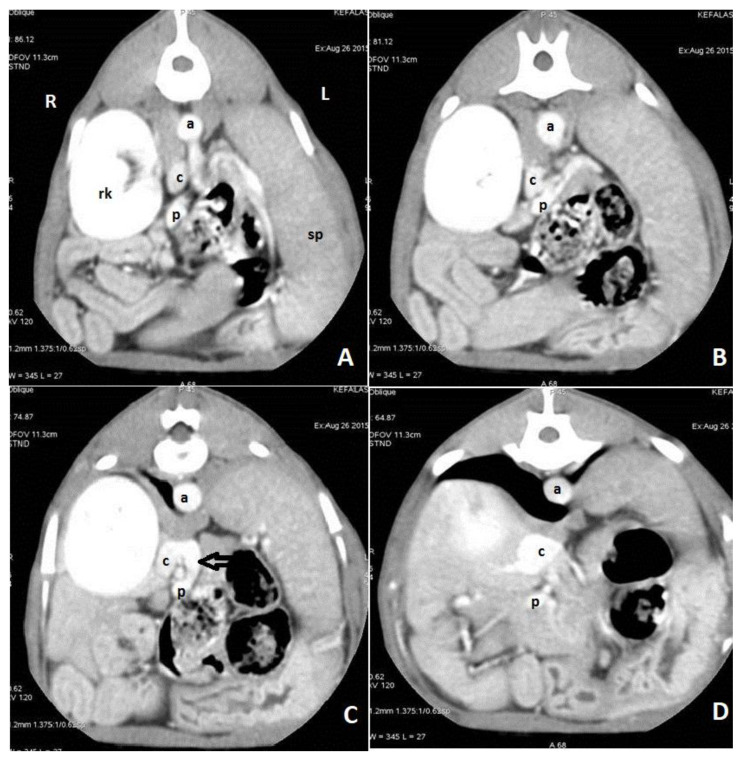
Axial portal phase angiographic images of a dog with an extrahepatic portocaval shunt. Images are ordered from caudal to cranial (**A**–**D**). A shunt vessel (black arrow) arising from the portal vein (p) enters into the caudal vena cava (c). There is dilation of the caudal vena cava after the insertion of the shunt vessel. (a: aorta; rk: right kidney; sp: spleen: R: right side; L: left side).

**Figure 8:**
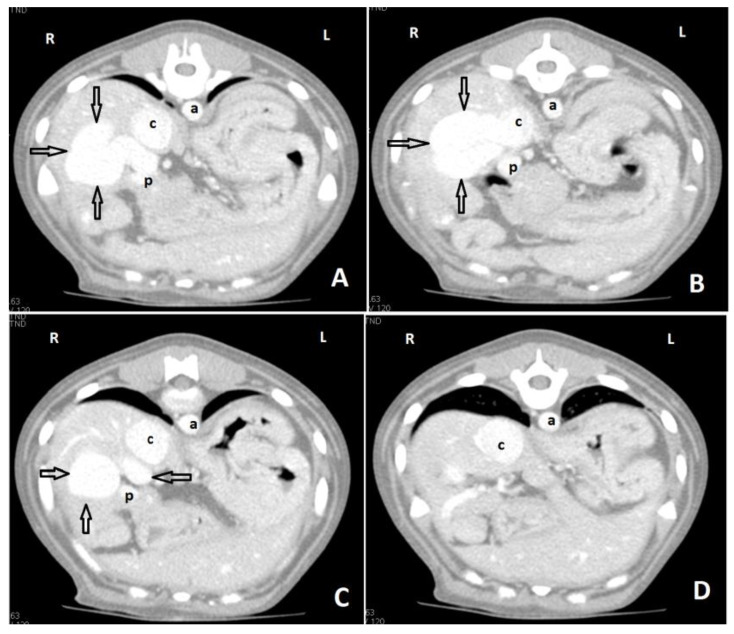
Axial portal phase angiographic images of a young dog with an intrahepatic portocaval shunt. Images are ordered from caudal to cranial (**A**–**D**). There is a large, tortuous shunt vessel (arrows) arising from the right side of the portal vein (p), and through the right liver lobes to enter the caudal vena cava (c). There is dilation of the caudal vena cava after the insertion of the shunt vessel. (a: aorta; R: right side; L: left side).

**Figure 9:**
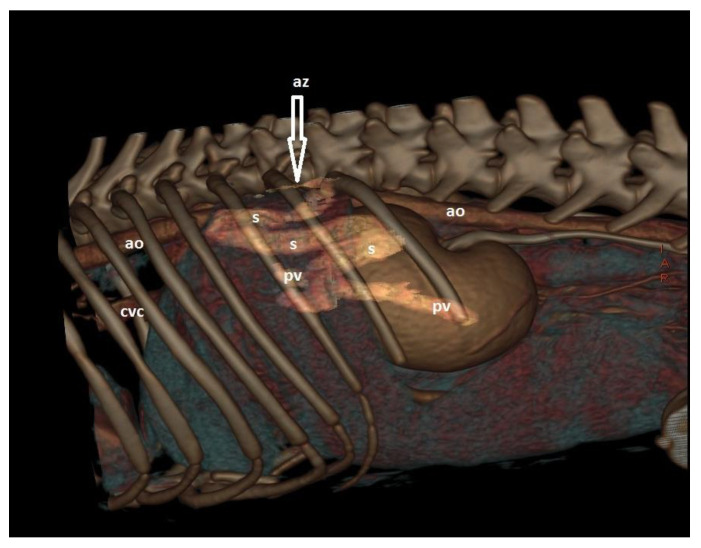
A 3D rendering image from the lateral aspect of a dog with a porto azygos shunt. There is a large, anomalous vessel (s) arising from the portal vein and after a tortous course travels dorsally and enters into the azygos vein (az). (ao: aorta; cvc: caudal vena cava; pv: portal vein).

CTA is a non-invasive, fast, accurate, and reasonably priced imaging technique that provides highly detailed information on shunt morphology and hepatic vasculature, and surgical planning [15,20,22,132,133,134,135]. CTA has become the imaging technique of choice for the diagnosis of CPSS in dogs and cats [Figure 7 and Figure 8 ], as it offers great sensitivity and specificity for the detection of CPSS [135]. Kim et al. (2013) reported that the sensitivity and specificity of CTA for the detection of PSS (96% and 89%, respectively) were significantly higher than those of abdominal ultrasonography (68% and 84%, respectively), and CTA was 5.5 times more likely to determine the presence of a shunt than abdominal ultrasound [132]. Following the completion of the study, further manipulation and reconstruction of the images are possible (3-dimensional images) using appropriate software [Figure 9]. The authors recommend to always perform a CTA, if available, to reach a definitive diagnosis, especially if a CPSS is highly suspected and cannot be located using US, or if a complex or multiple CPSS is suspected.

Magnetic resonance angiography (MRA) provides excellent 3-dimensional imaging of the CPSS and valuable presurgical planning. Several studies report the use of MRA for the detection of CPSS [136,137,138]. Seguin et al. (1999) reported sensitivity (80%) and specificity (100%) of MRA for the diagnosis of PSS comparable to those of CTA and differentiation of ECPSS from ICPSS was possible in the vast majority of dogs (83%) [137]. However, CTA is simpler, faster, and not so expensive compared to MRA.

## 10. Differential Diagnosis

The signalment, clinical signs, physical examination, and laboratory findings are often such that they sometimes strongly suggest CPSS. However, in any dog or cat suspected of having CPSS, diseases causing similar clinical signs such as gastrointestinal parasitism, toy breed juvenile hypoglycemia, idiopathic epilepsy, hydrocephalus, hypoadrenocorticism, protein-losing enteropathy, and enzyme deficiencies associated with the urea cycle should be also considered. In case of increased serum BA and plasma FA differentiating primary hypoplasia of the portal vein with (e.g., noncirrhotic portal hypertension [NCPH]) and without (formerly microvascular dysplasia [MVD]) portal hypertension (PHPV without PH), hepatic arteriovenous malformations (HAVMs), and CPSS is necessary. Dogs with other liver diseases such as chronic hepatitis, lobular dissecting hepatitis, cirrhosis, or even acute liver disease associated with leptospirosis can have similar clinical signs, but they are usually older and usually have hyperbilirubinemia. PHPV without PH is worthy of special mention as these dogs have a similar signalment to those with an ECPSS and share many clinicopathological features. PHPV(MVD) is also a congenital and probably linked-inherited vascular malformation associated with abnormal microscopic hepatic portal circulation [47,104,139,140]. PHPV without PH is characterized by reduced development of the intrahepatic terminal branches of the portal vein and as a consequence, the portal blood does not reach the hepatocytes. More specifically, the hypoplastic terminal intrahepatic portal venules cause increased arterial blood flow to maintain normal hepatic sinusoidal blood flow and the hepatic arteries become hyperplastic and torturous in the portal triads. These changes may result in sinusoidal hypertension, the opening of embryologic sinusoidal vessels, and blood transportation to central veins leading to hepatic atrophy associated with abnormal perfusion of the hepatic parenchyma and reduced supply of trophic factors. CPSS and PHPV without PH are genetically related disorders and PHPV without PH can occur with or without concurrent CPSS. PHPV(MVD) was first described in Cairn Terries [140]. Many small-breed dogs, such as Cairn Terriers, Yorkshire terriers, Maltese, Tibetan spaniels, miniature Schnauzer, Shih Tzu, and miniature and toy Poodles, are at increased risk for PHPV(MVD) [139]. There is no sex predisposition, although in a retrospective study (Christiansen et al. 2000) female dogs were seen more frequently than males (70% of the cases) [139]. The average age at presentation of PHPV(MVD) dogs is significantly higher than that of CPSS dogs, possibly due to less severe clinical signs or no (incidental high serum bile acid concentrations) [139]. Dogs with PHPV(MVD) can be divided into two groups, symptomatic and asymptomatic group. Most PHPV without PH dogs is asymptomatic and usually do not have the clinical signs seen in dogs with CPSS. Some of them may exhibit drug intolerance to substances metabolized or extracted by the liver, often noticed at the time of neutering. However, there are reports of PHPV without PH dogs having CNS, gastrointestinal and urinary signs [104,139]. The authors’ experience is that the vast majority of dogs with PHPV without PH are asymptomatic. It is possible that at least some symptomatic dogs with PHPV without PH may represent CPSS cases, where the shunt escaped diagnosis or clinical signs severity is related to the number of liver lobes affected.

## 11. Conclusions

Signalment, physical and clinicopathological findings can help raise suspicion of CPSS. However, they are non-specific and may wax and wane. Diagnosis is best achieved by coupling a liver function test (serum BA and/or NH_3_ concentrations) to a liver imaging technique such as US and/or CTA. CTA has become the imaging technique of choice for the diagnosis of CPSS as it provides surgical planning.

## Figures and Tables

**Table 1:** Anatomic classification of congenital portosystemic shunts in dogs.

Anatomic Classification of Extrahepatic Congenital Portosystemic Shunts in Dogs *	
Splenophrenic	
Splenoazygos	
Right gastro-caval	
Splenocaval	
Right gastrocaval with a caudal loop	
Gastrophrenic	
Left gastroazygos	
Colonocaval	
Portocaval	
Anatomic classification of intrahepatic congenital portosystemic shunts in dogs	
Traditional anatomic classification of intrahepatic congenital portosystemic shunts in dogs **	
Right divisional	
Left divisional	
Central divisional	
Anatomic subclassification of intrahepatic congenital portosystemic shunts in dogs **	
Shunt Type	Insertion
Right divisional	Right lateral hepatic vein
	Caudate hepatic vein
Left divisional	Left hepatic vein
	Left phrenic vein
Central divisional	Quadrate hepatic vein
	Central hepatic vein
	Dorsal right medial hepatic vein
	Ventral aspect of caudal vena cava
Multiple	Variable
Anatomic classification of intrahepatic congenital portosystemic shunts in dogs ***	
Left, right, central divisional Aneurysmal intrahepatic congenital portosystemic shunts One or more portosystemic shunts in a single liver lobe Multiple portosystemic shunts in several liver lobes	

* White et al. (2018) and Fukushima et al. (2014) [14,15]. ** Plested et al. (2020) [19]. *** Bertolini et al. (2019) [18].

**Table 2:** Veterinary modification of West Haven grading scale for hepatic encephalopathy.

Grade	Clinical Signs
0	Asymptomatic
I	Mild decrease in mobility, apathy, or both
II	Severe apathy, mild ataxia
III	Hypersalivation, severe ataxia, head pressing, blindness, circling
IV	Seizures, stupors, or coma

**Table 3:** Precipitating factors for hepatic encephalopathy in dogs.

Precipitating Factors
Gastrointestinal hemorrhage
Transfusion of stored blood
High-protein diet
Constipation
Hypokalemia
Hyponatremia
Metabolic Alkalosis
Dehydration
Infection
Sepsis
Various Drugs (e.g., diuretics, opioids)
Non-compliance with medical treatment

## Data Availability

Not applicable.

## References

[1] Tobias K., Slatter D. (2003). Portosystemic Shunts and Other Hepatic Vascular Anomalies. Textbook of Small Animal Surgery.

[2] Berent A., Tobias K., Johnston S., Tobias K. (2018). Hepatic Vascular Anomalies. Veterinary Surgery: Small Animal.

[3] Weisse C., Berent A., Ettinger S., Feldman E., Cote E. (2017). Hepatic Vascular Anomalies. Textbook of Veterinary Internal Medicine.

[4] Markowitz J., Rappaport A., Scott A.C. (1949). The Function of the Hepatic Artery in the Dog. Am. J. Dig. Dis..

[5] Evans H.E., de Lahunta A., Evans H.E., de Lahunta A. (2013). The Digestive Apparatus and Abdomen. Miller’s Anatomy of the Dog.

[6] Washabau R.J., Washabau R.J., Day M.J. (2013). Liver. Canine and Feline Gastroenterology.

[7] Hickman J., Edwards J.E., Mann F. (1949). Venous Anomalies in a Dog; Absence of the Portal Vein; Continuity of Lower Part of Inferior Vena Cava with the Azygos Vein. Anat. Rec..

[8] Audell L., Jönsson L., Lannek B. (1974). Congenital Porta-Caval Shunts in the Dog; a Description of Three Cases. Zentralbl. Veterinarmed. A.

[9] Ewing G., Suter P., Bailey C. (1974). Hepatic Insufficiency Associated with Congenital Anomalies of the Portal Vein in Dogs. J. Am. Anim. Hosp. Assoc..

[10] White R.N., Parry A.T. (2016). Morphology of Splenocaval Congenital Portosystemic Shunts in Dogs and Cats. J. Small Anim. Pract..

[11] White R.N., Parry A.T. (2015). Morphology of Congenital Portosystemic Shunts Involving the Right Gastric Vein in Dogs. J. Small Anim. Pract..

[12] White R.N., Parry A.T. (2016). Morphology of Congenital Portosystemic Shunts Involving the Left Colic Vein in Dogs and Cats. J. Small Anim. Pract..

[13] White R.N., Parry A.T. (2013). Morphology of Congenital Portosystemic Shunts Emanating from the Left Gastric Vein in Dogs and Cats. J. Small Anim. Pract..

[14] White R.N., Parry A.T., Shales C. (2018). Implications of Shunt Morphology for the Surgical Management of Extrahepatic Portosystemic Shunts. Aust. Vet. J..

[15] Fukushima K., Kanemoto H., Ohno K., Takahashi M., Fujiwara R., Nishimura R., Tsujimoto H. (2014). Computed Tomographic Morphology and Clinical Features of Extrahepatic Portosystemic Shunts in 172 Dogs in Japan. Vet. J..

[16] White R.N., Shales C., Parry A.T. (2017). New Perspectives on the Development of Extrahepatic Portosystemic Shunts. J. Small Anim. Pract..

[17] Seller S., Weisse C., Fischetti A.J. (2022). Intrahepatic Venous Collaterals in Dogs with Congenital Intrahepatic Portosystemic Shunts Are Associated with Focal Shunt or Hepatic Vein Narrowing. Vet. Radiol. Ultrasound.

[18] Bertolini G. (2019). Anomalies of the Portal Venous System in Dogs and Cats as Seen on Multidetector-Row Computed Tomography: An Overview and Systematization Proposal. Vet. Sci..

[19] Plested M.J., Zwingenberger A.L., Brockman D.J., Hecht S., Secrest S., Culp W.T.N., Drees R. (2020). Canine Intrahepatic Portosystemic Shunt Insertion into the Systemic Circulation Is Commonly through Primary Hepatic Veins as Assessed with CT Angiography. Vet. Radiol. Ultrasound.

[20] Frank P., Mahaffey M., Egger C., Cornell K.K. (2003). Helical Computed Tomographic Portography in Ten Normal Dogs and Ten Dogs with a Portosystemic Shunt. Vet. Radiol. Ultrasound.

[21] Bertolini G., Diana A., Cipone M., Drigo M., Caldin M. (2014). Multidetector Row Computed Tomography and Ultrasound Characteristics of Caudal Vena Cava Duplication in Dogs. Vet. Radiol. Ultrasound.

[22] Zwingenberger A.L., Schwarz T., Saunders H.M. (2005). Helical Computed Tomographic Angiography of Canine Portosystemic Shunts. Vet. Radiol. Ultrasound.

[23] White R.N., Burton C.A., McEvoy F.J. (1998). Surgical Treatment of Intrahepatic Portosystemic Shunts in 45 Dogs. Vet. Rec..

[24] Lamb C.R., White R.N. (1998). Morphology of Congenital Intrahepatic Portacaval Shunts in Dogs and Cats. Vet. Rec..

[25] Parry A.T., White R.N. (2017). Comparison of Computed Tomographic Angiography and Intraoperative Mesenteric Portovenography for Extrahepatic Portosystemic Shunts. J. Small Anim. Pract..

[26] White R.N., Burton C.A. (2000). Anatomy of the Patent Ductus Venosus in the Dog. Vet. Rec..

[27] Payne J.T., Martin R.A., Constantinescu G.M. (1990). The Anatomy and Embryology of Portosystemic Shunts in Dogs and Cats. Semin. Vet. Med. Surg. Small Anim..

[28] Lamb C.R., Burton C.A. (2004). Doppler Ultrasonographic Assessment of Closure of the Ductus Venosus in Neonatal Irish Wolfhounds. Vet. Rec..

[29] Strickland R., Tivers M.S., Adamantos S.E., Harcourt-Brown T.R., Fowkes R.C., Lipscomb V.J. (2018). Incidence and Risk Factors for Neurological Signs after Attenuation of Single Congenital Portosystemic Shunts in 253 Dogs. Vet. Surg..

[30] White R.N., Forster-van Hijfte M.A., Petrie G., Lamb C.R., Hammond R.A. (1996). Surgical Treatment of Intrahepatic Portosystemic Shunts in Six Cats. Vet. Rec..

[31] Tivers M., Lipscomb V. (2011). Congenital Portosystemic Shunts in Cats: Investigation, Diagnosis and Stabilisation. J. Feline Med. Surg..

[32] Leeman J.J., Kim S.E., Reese D.J., Risselada M., Ellison G.W. (2013). Multiple Congenital PSS in a Dog: Case Report and Literature Review. J. Am. Anim. Hosp. Assoc..

[33] Gow A.G. (2017). Hepatic Encephalopathy. Vet. Clin. N. Am. Small Anim. Pract..

[34] Lidbury J.A., Cook A.K., Steiner J.M. (2016). Hepatic Encephalopathy in Dogs and Cats. J. Vet. Emerg. Crit. Care.

[35] Ferenci P., Lockwood A., Mullen K., Tarter R., Weissenborn K., Blei A.T. (2002). Hepatic Encephalopathy—Definition, Nomenclature, Diagnosis, and Quantification: Final Report of the Working Party at the 11th World Congresses of Gastroenterology, Vienna, 1998. Hepatology.

[36] Szatmári V., Rothuizen J., van den Ingh T.S.G.M., van Sluijs F.J., Voorhout G. (2004). Ultrasonographic Findings in Dogs with Hyperammonemia: 90 Cases (2000–2002). J. Am. Vet. Med. Assoc..

[37] Proot S., Biourge V., Teske E., Rothuizen J. (2009). Soy Protein Isolate versus Meat-Based Low-Protein Diet for Dogs with Congenital Portosystemic Shunts. J. Vet. Intern. Med..

[38] Jalan R., Shawcross D., Davies N. (2003). The Molecular Pathogenesis of Hepatic Encephalopathy. Int. J. Biochem. Cell Biol..

[39] Keiding S., Sørensen M., Bender D., Munk O.L., Ott P., Vilstrup H. (2006). Brain Metabolism of 13N-Ammonia during Acute Hepatic Encephalopathy in Cirrhosis Measured by Positron Emission Tomography. Hepatology.

[40] Bhatia V., Singh R., Acharya S.K. (2006). Predictive Value of Arterial Ammonia for Complications and Outcome in Acute Liver Failure. Gut.

[41] Rothuizen J., van den Ingh T.S.G.A.M. (1982). Arterial and Venous Ammonia Concentrations in the Diagnosis of Canine Hepato-Encephalopathy. Res. Vet. Sci..

[42] Shawcross D., Jalan R. (2005). The Pathophysiologic Basis of Hepatic Encephalopathy: Central Role for Ammonia and Inflammation. Cell. Mol. Life Sci..

[43] Coltart I., Tranah T.H., Shawcross D.L. (2013). Inflammation and Hepatic Encephalopathy. Arch. Biochem. Biophys..

[44] Gow A.G., Marques A.I., Yool D.A., Crawford K., Warman S.M., Eckersall P.D., Jalan R., Mellanby R.J. (2012). Dogs with Congenital Porto-Systemic Shunting (CPSS) and Hepatic Encephalopathy Have Higher Serum Concentrations of C-Reactive Protein than Asymptomatic Dogs with CPSS. Metab. Brain Dis..

[45] Tivers M.S., Handel I., Gow A.G., Lipscomb V.J., Jalan R., Mellanby R.J. (2014). Hyperammonemia and Systemic Inflammatory Response Syndrome Predicts Presence of Hepatic Encephalopathy in Dogs with Congenital Portosystemic Shunts. PLoS ONE.

[46] Howe L.M., Boothe D.M., Boothe H.W. (1997). Endotoxemia Associated with Experimentally Induced Multiple Portosystemic Shunts in Dogs. Am. J. Vet. Res..

[47] Cullen J., van den Ingh T., Bunch S., Rothuizen J., Washabau R., Desmet V., WSAVA Liver Standardization Group (2006). Morphological Classification of Circulatory Disorders of the Canine and Feline Liver. WSAVA Standards for Clinical and Histological Diagnosis of Canine and Feline Liver Diseases.

[48] Kelley D., Lester C., Delaforcade A., Webster C.R.L. (2013). Thromboelastographic Evaluation of Dogs with Congenital Portosystemic Shunts. J. Vet. Intern. Med..

[49] Kummeling A., Teske E., Rothuizen J., van Sluijs F.J. (2006). Coagulation Profiles in Dogs with Congenital Portosystemic Shunts before and after Surgical Attenuation. J. Vet. Intern. Med..

[50] Hunt G.B., Kummeling A., Tisdall P.L.C., Marchevsky A.M., Liptak J.M., Youmans K.R., Goldsmid S.E., Beck J.A. (2004). Outcomes of Cellophane Banding for Congenital Portosystemic Shunts in 106 Dogs and 5 Cats. Vet. Surg..

[51] Mehl M.L., Kyles A.E., Hardie E.M., Kass P.H., Adin C., Flynn A.K., De Cock H.E., Gregory C.R. (2005). Evaluation of Ameroid Ring Constrictors for Treatment for Single Extrahepatic Portosystemic Shunts in Dogs: 168 Cases (1995–2001). J. Am. Vet. Med. Assoc..

[52] Holford A.L., Tobias K.M., Bartges J.W., Johnson B.M. (2008). Adrenal Response to Adrenocorticotropic Hormone in Dogs before and after Surgical Attenuation of a Single Congenital Portosystemic Shunt. J. Vet. Intern. Med..

[53] Tobias K.M., Rohrbach B.W. (2003). Association of Breed with the Diagnosis of Congenital Portosystemic Shunts in Dogs: 2400 Cases (1980–2002). J. Am. Vet. Med. Assoc..

[54] Weisse C., Berent A.C., Todd K., Solomon J.A., Cope C. (2014). Endovascular Evaluation and Treatment of Intrahepatic Portosystemic Shunts in Dogs: 100 Cases (2001–2011). J. Am. Vet. Med. Assoc..

[55] Bostwick D.R., Twedt D.C. (1995). Intrahepatic and Extrahepatic Portal Venous Anomalies in Dogs: 52 Cases (1982–1992). J. Am. Vet. Med. Assoc..

[56] Lamb C.R., Forster-van Hijfte M.A., White R.N., McEvoy F.J., Rutgers H.C. (1996). Ultrasonographic Diagnosis of Congenital Portosystemic Shunt in 14 Cats. J. Small Anim. Pract..

[57] Rothuizen J., van den Ingh T.S.G.A.M., Voorhoutm G., van dER Luer R.J.T., Wouda W. (1982). Congenital Portosystemic Shunts in Sixteen Dogs and Three Cats. J. Small Anim. Pract..

[58] Hunt G.B. (2004). Effect of Breed on Anatomy of Portosystemic Shunts Resulting from Congenital Diseases in Dogs and Cats: A Review of 242 Cases. Aust. Vet. J..

[59] Tillson D.M., Winkler J.T. (2002). Diagnosis and Treatment of Portosystemic Shunts in the Cat. Vet. Clin. N. Am.-Small Anim. Pract..

[60] Blaxter A.C., Holt P.E., Pearson G.R., Gibbs C., Gruffydd-Jones T.J. (1988). Congenital Portosystemic Shunts in the Cat: A Report of Nine Cases. J. Small Anim. Pract..

[61] van Steenbeek F.G., Leegwater P.A.J., van Sluijs F.J., Heuven H.C.M., Rothuizen J. (2009). Evidence of Inheritance of Intrahepatic Portosystemic Shunts in Irish Wolfhounds. J. Vet. Intern. Med..

[62] Kerr M.G., Van Doorn T. (1999). Mass Screening of Irish Wolfhound Puppies for Portosystemic Shunts by the Dynamic Bile Acid Test. Vet. Rec..

[63] Meyer H.P., Rothuizen J., Ubbink G.J., van den Ingh T.S. (1995). Increasing Incidence of Hereditary Intrahepatic Portosystemic Shunts in Irish Wolfhounds in The Netherlands (1984 to 1992). Vet. Rec..

[64] Worley D.R., Holt D.E. (2008). Clinical Outcome of Congenital Extrahepatic Portosystemic Shunt Attenuation in Dogs Aged Five Years and Older: 17 Cases (1992–2005). J. Am. Vet. Med. Assoc..

[65] Winkler J.T., Bohling M.W., Tillson M.D., Wright J.C., Ballagas A.J. (2003). Portosystemic Shunts: Diagnosis, Prognosis, and Treatment of 64 Cases (1993–2001). J. Am. Anim. Hosp. Assoc..

[66] Boothe H.W., Howe L.M., Edwards J.F., Slater M.R. (1996). Multiple Extrahepatic Portosystemic Shunts in Dogs: 30 Cases (1981–1993). J. Am. Vet. Med. Assoc..

[67] Fryer K.J., Levine J.M., Peycke L.E., Thompson J.A., Cohen N.D. (2011). Incidence of Postoperative Seizures with and without Levetiracetam Pretreatment in Dogs Undergoing Portosystemic Shunt Attenuation. J. Vet. Intern. Med..

[68] van den Ingh T.S.G.A.M., Rothuizen J., Meyer H.P. (1995). Circulatory Disorders of the Liver in Dogs and Cats. Vet. Q..

[69] Berent A.C., Tobias K.M. (2009). Portosystemic Vascular Anomalies. Vet. Clin. N. Am. Small Anim. Pract..

[70] Havig M., Tobias K.M. (2002). Outcome of Ameroid Constrictor Occlusion of Single Congenital Extrahepatic Portosystemic Shunts in Cats: 12 Cases (1993–2000). J. Am. Vet. Med. Assoc..

[71] Lipscomb V.J., Jones H.J., Brockman D.J. (2007). Complications and Long-Term Outcomes of the Ligation of Congenital Portosystemic Shunts in 49 Cats. Vet. Rec..

[72] Caporali E.H.G., Phillips H., Underwood L., Selmic L.E. (2015). Risk Factors for Urolithiasis in Dogs with Congenital Extrahepatic Portosystemic Shunts: 95 Cases (1999–2013). J. Am. Vet. Med. Assoc..

[73] Dear J.D., Shiraki R., Ruby A.L., Westropp J.L. (2011). Feline Urate Urolithiasis: A Retrospective Study of 159 Cases. J. Feline Med. Surg..

[74] van Gundy T.E., Boothe H.W., Wolf A. (1990). Results of Surgical Management of Feline Portosystemic Shunts. J. Am. Anim. Hosp. Assoc..

[75] Lipscomb V.J., Lee K.C., Lamb C.R., Brockman D.J. (2009). Association of Mesenteric Portovenographic Findings with Outcome in Cats Receiving Surgical Treatment for Single Congenital Portosystemic Shunts. J. Am. Vet. Med. Assoc..

[76] Lamb C. (1996). Ultrasonographic Diagnosis of Congenital Portosystemic Shunts on Dogs: Results of a Prospective Study. Vet. Radiol. Ultrasound.

[77] Kyles A.E., Hardie E.M., Mehl M., Gregory C.R. (2002). Evaluation of Ameroid Ring Constrictors for the Management of Single Extrahepatic Portosystemic Shunts in Cats: 23 Cases (1996–2001). J. Am. Vet. Med. Assoc..

[78] Deppe T.A., Center S.A., Simpson K.W., Erb H.N., Randolph J.F., Dykes N.L., Yeager A.E., Reynolds A.J. (1999). Glomerular Filtration Rate and Renal Volume in Dogs with Congenital Portosystemic Vascular Anomalies before and after Surgical Ligation. J. Vet. Intern. Med..

[79] Scavelli T.D., Hornbuckle W.E., Roth L., Rendano V.T., de Lahunta A., Center S.A., French T.W., Zimmer J.F. (1986). Portosystemic Shunts in Cats: Seven Cases (1976-1984). J. Am. Vet. Med. Assoc..

[80] Kraun M.B., Nelson L.L., Hauptman J.G., Nelson N.C. (2014). Analysis of the Relationship of Extrahepatic Portosystemic Shunt Morphology with Clinical Variables in Dogs: 53 Cases (2009–2012). J. Am. Vet. Med. Assoc..

[81] Sura P.A., Tobias K.M., Morandi F., Daniel G.B., Echandi R.L. (2007). Comparison of 99mTcO4(-) Trans-Splenic Portal Scintigraphy with per-Rectal Portal Scintigraphy for Diagnosis of Portosystemic Shunts in Dogs. Vet. Surg..

[82] Simpson K.W., Meyer D.J., Boswood A., White R.N., Maskell I.E. (1997). Iron Status and Erythrocyte Volume in Dogs with Congenital Portosystemic Vascular Anomalies. J. Vet. Intern. Med..

[83] Bunch S.E., Jordan H.L., Sellon R.K., Cullen J.M., Smith J.E. (1995). Characterization of Iron Status in Young Dogs with Portosystemic Shunt. Am. J. Vet. Res..

[84] Laflamme D.P., Mahaffey E.A., Allen S.W., Twedt D.C., Prasse K.W., Huber T.L. (1994). Microcytosis and Iron Status in Dogs With Surgically Induced Portosystemic Shunts. J. Vet. Intern. Med..

[85] Frowde P.E., Gow A.G., Burton C.A., Powell R., Lipscomb V.J., House A.K., Mellanby R.J., Tivers M.S. (2014). Hepatic Hepcidin Gene Expression in Dogs with a Congenital Portosystemic Shunt. J. Vet. Intern. Med..

[86] Watson P.J., Herrtage M.E. (1998). Medical Management of Congenital Portosystemic Shunts in 27 Dogs-a Retrospective Study. J. Small Anim. Pract..

[87] Papazoglou L.G., Monnet E., Seim H.B. (2002). Survival and Prognostic Indicators for Dogs with Intrahepatic Portosystemic Shunts: 32 Cases (1990–2000). Vet. Surg..

[88] Webster C.R.L. (2017). Hemostatic Disorders Associated with Hepatobiliary Disease. Vet. Clin. N. Am. Small Anim. Pract..

[89] Toulza O., Center S., Brooks M.B., Erb H.N., Warner K.L., Deal W. (2006). Evaluation of Plasma Protein C Activity for Detection of Hepatobiliary Disease and Portosystemic Shunting in Dogs. J. Am. Vet. Med. Assoc..

[90] Niles J.D., Williams J.M., Cripps P.J. (2001). Hemostatic Profiles in 39 Dogs with Congenital Portosystemic Shunts. Vet. Surg..

[91] Prins M., Schellens C.J.M.M., van Leeuwen M.W., Rothuizen J., Teske E. (2010). Coagulation Disorders in Dogs with Hepatic Disease. Vet. J..

[92] Roy R.G., Post G.S., Waters D.J., Hardy R.M. (1992). Portal Vein Thrombosis as a Complication of Portosystemic Shunt Ligation in Two Dogs. J Am Anim. Hosp. Assoc..

[93] Tzounos C.E., Tivers M.S., Adamantos S.E., English K., Rees A.L., Lipscomb V.J. (2017). Haematology and Coagulation Profiles in Cats with Congenital Portosystemic Shunts. J. Feline Med. Surg..

[94] Center S.A., Magne M.L. (1990). Historical, Physical Examination, and Clinicopathologic Features of Portosystemic Vascular Anomalies in the Dog and Cat. Semin. Vet. Med. Surg. (Small Anim.).

[95] Broome C.J., Walsh V.P., Braddock J. (2004). a Congenital Portosystemic Shunts in Dogs and Cats. N. Z. Vet. J..

[96] Johnson C.A., Armstrong P.J., Hauptman J.G. (1987). Congenital Portosystemic Shunts in Dogs: 46 Cases (1979–1986). J. Am. Vet. Med. Assoc..

[97] Center S.A., ManWarren T., Slater M.R., Wilentz E. (1991). Evaluation of Twelve-Hour Preprandial and Two-Hour Postprandial Serum Bile Acids Concentrations for Diagnosis of Hepatobiliary Disease in Dogs. J. Am. Vet. Med. Assoc..

[98] Center S.A., Erb H.N., Joseph S.A. (1995). Measurement of Serum Bile Acids Concentrations for Diagnosis of Hepatobiliary Disease in Cats. J. Am. Vet. Med. Assoc..

[99] Center S.A., Baldwin B.H., Erb H., Tennant B.C. (1986). Bile Acid Concentrations in the Diagnosis of Hepatobiliary Disease in the Cat. J. Am. Vet. Med. Assoc..

[100] Center S.A., Baldwin B.H., de Lahunta A., Dietze A.E., Tennant B.C. (1985). Evaluation of Serum Bile Acid Concentrations for the Diagnosis of Portosystemic Venous Anomalies in the Dog and Cat. J. Am. Vet. Med. Assoc..

[101] Ruland K., Fischer A., Hartmann K. (2010). Sensitivity and Specificity of Fasting Ammonia and Serum Bile Acids in the Diagnosis of Portosystemic Shunts in Dogs and Cats. Vet. Clin. Pathol..

[102] Jensen A.L. (1991). Evaluation of Fasting and Postprandial Total Serum Bile Acid Concentration in Dogs with Hepatobiliary Disorders. Zentralbl. Veterinarmed. A.

[103] Chapman S.E., Hostutler R.A. (2013). A Laboratory Diagnostic Approach to Hepatobiliary Disease in Small Animals. Vet. Clin. N. Am. Small Anim. Pract..

[104] Allen L., Stobie D., Mauldin G.N., Baer K.E. (1999). Clinicopathologic Features of Dogs with Hepatic Microvascular Dysplasia with and without Portosystemic Shunts: 42 Cases (1991–1996). J. Am. Vet. Med. Assoc..

[105] Tisdall P.L., Hunt G.B., Bellenger C.R., Malik R. (1994). Congenital Portosystemic Shunts in Maltese and Australian Cattle Dogs. Aust. Vet. J..

[106] Deitz K.L., Makielski K.M., Williams J.M., Lin H., Morrison J.A. (2015). Effect of 6-8 Weeks of Oral Ursodeoxycholic Acid Administration on Serum Concentrations of Fasting and Postprandial Bile Acids and Biochemical Analytes in Healthy Dogs. Vet. Clin. Pathol..

[107] Strombeck D.R., Meyer D.J., Freedland R.A. (1975). Hyperammonemia Due to a Urea Cycle Enzyme Deficiency in Two Dogs. J. Am. Vet. Med. Assoc..

[108] Zandvliet M.M.J.M., Rothuizen J. (2007). Transient Hyperammonemia Due to Urea Cycle Enzyme Deficiency in Irish Wolfhounds. J. Vet. Intern. Med..

[109] Walker M.C., Hill R.C., Guilford W.G., Scott K.C., Jones G.L., Buergelt C.D. (2001). Postprandial Venous Ammonia Concentrations in the Diagnosis of Hepatobiliary Disease in Dogs. J. Vet. Intern. Med..

[110] Whiting P.G., Breznock E.M., Moore P., Kerr L., Berger B., Gregory C., Hornof W. (1986). Partial Hepatectomy with Temporary Hepatic Vascular Occlusion in Dogs with Hepatic Arteriovenous Fistulas. Vet. Surg..

[111] van Straten G., Spee B., Rothuizen J., van Straten M., Favier R.P. (2015). Diagnostic Value of the Rectal Ammonia Tolerance Test, Fasting Plasma Ammonia and Fasting Plasma Bile Acids for Canine Portosystemic Shunting. Vet. J..

[112] Rothuizen J., van den Ingh T.S.G.A.M. (1982). Rectal Ammonia Tolerance Test in the Evaluation of Portal Circulation in Dogs with Liver Disease. Res. Vet. Sci..

[113] Meyer H.P., Rothuizen J., Tiemessen I., Van Den Brom W.E., Van Den Ingh T.S.G.A.M. (1996). Transient Metabolic Hyperammonaemia in Young Irish Wolfhounds. Vet. Rec..

[114] Danese S., Vetrano S., Zhang L., Poplis V.A., Castellino F.J. (2010). The Protein C Pathway in Tissue Inflammation and Injury: Pathogenic Role and Therapeutic Implications. Blood.

[115] Aird W.C. (2004). Natural Anticoagulant Inhibitors: Activated Protein C. Best Pract. Res. Clin. Haematol..

[116] Sunlight C., Weisse C., Berent A., Tozier E. (2022). Protein C and Comparative Biochemical Changes in Dogs Treated with Percutaneous Transvenous Coil Embolization of Congenital Intrahepatic Portosystemic Shunts. Vet. Surg..

[117] Tarnow I., Falk T., Tidholm A., Martinussen T., Jensen A.L., Olsen L.H., Pedersen H.D., Kristensen A.T. (2007). Hemostatic Biomarkers in Dogs with Chronic Congestive Heart Failure. J. Vet. Intern. Med..

[118] de Laforcade A.M., Rozanski E.A., Freeman L.M., Li W. (2008). Serial Evaluation of Protein C and Antithrombin in Dogs with Sepsis. J. Vet. Intern. Med..

[119] de Laforcade A.M., Freeman L.M., Shaw S.P., Brooks M.B., Rozanski E.A., Rush J.E. (2003). Hemostatic Changes in Dogs with Naturally Occurring Sepsis. J. Vet. Intern. Med..

[120] Lee K.C.L., Winstanley A., House J.V., Lipscomb V., Lamb C., Gregory S., Jalan R., Mookerjee R.P., Brockman D.J. (2011). Association between Hepatic Histopathologic Lesions and Clinical Findings in Dogs Undergoing Surgical Attenuation of a Congenital Portosystemic Shunt: 38 Cases (2000–2004). J. Am. Vet. Med. Assoc..

[121] Parker J.S., Monnet E., Powers B.E., Twedt D.C. (2008). Histologic Examination of Hepatic Biopsy Samples as a Prognostic Indicator in Dogs Undergoing Surgical Correction of Congenital Portosystemic Shunts: 64 Cases (1997–2005). J. Am. Vet. Med. Assoc..

[122] Isobe K., Matsunaga S., Nakayama H., Uetsuka K. (2008). Histopathological Characteristics of Hepatic Lipogranulomas with Portosystemic Shunt in Dogs. J. Vet. Med. Sci. Jpn. Soc. Vet. Sci..

[123] Baade S., Aupperle H., Grevel V., Schoon H.A. (2006). Histopathological and Immunohistochemical Investigations of Hepatic Lesions Associated with Congenital Portosystemic Shunt in Dogs. J. Comp. Pathol..

[124] Swinbourne F., Smith K.C., Lipscomb V.J., Tivers M.S. (2013). Histopathological Findings in the Livers of Cats with a Congenital Portosystemic Shunt before and after Surgical Attenuation. Vet. Rec..

[125] Hunt G.B., Luff J., Daniel L., Zwingenberger A. (2014). Does Hepatic Steatosis Have an Impact on the Short Term Hepatic Response after Complete Attenuation of Congenital Extrahepatic Portosystemic Shunts? A Prospective Study of 20 Dogs. Vet. Surg..

[126] Lamb C.R. (1998). Ultrasonography of Portosystemic Shunts in Dogs and Cats. Vet. Clin. N. Am. Small Anim. Pract..

[127] Tiemessen I., Rothuizen J., Voorhout G. (1995). Ultrasonography in the Diagnosis of Congenital Portosystemic Shunts in Dogs. Vet. Q..

[128] D’Anjou M.A., Penninck D., Cornejo L., Pibarot P. (2004). Ultrasonographic Diagnosis of Portosystemic Shunting in Dogs and Cats. Vet. Radiol. Ultrasound.

[129] Szatmári V., Rothuizen J., Voorhout G. (2004). Standard Planes for Ultrasonographic Examination of the Portal System in Dogs. J. Am. Vet. Med. Assoc..

[130] Holt D.E., Schelling C.G., Saunders H.M., Orsher R.J. (1995). Correlation of Ultrasonographic Findings with Surgical, Portographic, and Necropsy Findings in Dogs and Cats with Portosystemic Shunts: 63 Cases (1987–1993). J. Am. Vet. Med. Assoc..

[131] Berent A., Weisse C., Ettinge S., Feldman E. (2010). Hepatic Vascular Anomalies. Textbook of Veterinary Internal Medicine.

[132] Kim S.E., Giglio R.F., Reese D.J., Reese S.L., Bacon N.J., Ellison G.W. (2013). Comparison of Computed Tomographic Angiography and Ultrasonography for the Detection and Characterization of Portosystemic Shunts in Dogs. Vet. Radiol. Ultrasound.

[133] Nelson N.C., Nelson L.L. (2011). Anatomy of Extrahepatic Portosystemic Shunts in Dogs as Determined by Computed Tomography Angiography. Vet. Radiol. Ultrasound.

[134] Zwingenberger A. (2009). CT Diagnosis of Portosystemic Shunts. Vet. Clin. N. Am.-Small Anim. Pract..

[135] Bertolini G., Rolla E.C., Zotti A., Caldin M. (2006). Three-Dimensional Multislice Helical Computed Tomography Techniques for Canine Extra-Hepatic Portosystemic Shunt Assessment. Vet. Radiol. Ultrasound.

[136] Mai W., Weisse C. (2011). Contrast-Enhanced Portal Magnetic Resonance Angiography in Dogs with Suspected Congenital Portal Vascular Anomalies. Vet. Radiol. Ultrasound.

[137] Seguin B., Tobias K.M., Gavin P.R., Tucker R.L. (1999). Use of Magnetic Resonance Angiography for Diagnosis of Portosystemic Shunts in Dogs. Vet. Radiol. Ultrasound.

[138] Bruehschwein A., Foltin I., Flatz K., Zoellner M., Matis U. (2010). Contrast-Enhanced Magnetic Resonance Angiography for Diagnosis of Portosystemic Shunts in 10 Dogs. Vet. Radiol. Ultrasound.

[139] Christiansen J.S., Hottinger H.A., Allen L., Phillips L., Aronson L.R. (2000). Hepatic Microvascular Dysplasia in Dogs: A Retrospective Study of 24 Cases (1987–1995). J. Am. Anim. Hosp. Assoc..

[140] Schermerhorn T., Center S.A., Dykes N.L., Rowland P.H., Yeager A.E., Erb H.N., Oberhansley K., Bonda M. (1996). Characterization of Hepatoportal Microvascular Dysplasia in a Kindred of Cairn Terriers. J. Vet. Intern. Med..

